# Ex Vivo Cardiotoxicity of Antineoplastic Casiopeinas Is Mediated through Energetic Dysfunction and Triggered Mitochondrial-Dependent Apoptosis

**DOI:** 10.1155/2018/8949450

**Published:** 2018-03-25

**Authors:** Christian Silva-Platas, César A. Villegas, Yuriana Oropeza-Almazán, Mariana Carrancá, Alejandro Torres-Quintanilla, Omar Lozano, Javier Valero-Elizondo, Elena C. Castillo, Judith Bernal-Ramírez, Evaristo Fernández-Sada, Luis F. Vega, Niria Treviño-Saldaña, Héctor Chapoy-Villanueva, Lena Ruiz-Azuara, Carmen Hernández-Brenes, Leticia Elizondo-Montemayor, Carlos E. Guerrero-Beltrán, Karla Carvajal, María E. Bravo-Gómez, Gerardo García-Rivas

**Affiliations:** ^1^Cátedra de Cardiología y Medicina Vascular, Escuela de Medicina, Tecnológico de Monterrey, 64849 Monterrey, NL, Mexico; ^2^Centro de Investigación Biomédica, Hospital Zambrano Hellion, Tecnológico de Monterrey, 66278 San Pedro Garza García, NL, Mexico; ^3^Departamento de Química Inorgánica, Facultad de Química, Universidad Nacional Autónoma de México, 04510 Mexico City, Mexico; ^4^Escuela de Ingeniería y Ciencias, Tecnológico de Monterrey, 64849 Monterrey, NL, Mexico; ^5^Laboratorio de Nutrición Experimental, Instituto Nacional de Pediatría, 04530 Mexico City, Mexico; ^6^Departamento de Toxicología, Facultad de Medicina, Universidad Nacional Autónoma de México, 04510 Mexico City, Mexico

## Abstract

Casiopeinas are a group of copper-based antineoplastic molecules designed as a less toxic and more therapeutic alternative to cisplatin or Doxorubicin; however, there is scarce evidence about their toxic effects on the whole heart and cardiomyocytes. Given this, rat hearts were perfused with Casiopeinas or Doxorubicin and the effects on mechanical performance, energetics, and mitochondrial function were measured. As well, the effects of Casiopeinas-triggered cell death were explored in isolated cardiomyocytes. Casiopeinas III-Ea, II-gly, and III-ia induced a progressive and sustained inhibition of heart contractile function that was dose- and time-dependent with an IC_50_ of 1.3 ± 0.2, 5.5 ± 0.5, and 10 ± 0.7 *μ*M, correspondingly. Myocardial oxygen consumption was not modified at their respective IC_50_, although ATP levels were significantly reduced, indicating energy impairment. Isolated mitochondria from Casiopeinas-treated hearts showed a significant loss of membrane potential and reduction of mitochondrial Ca^2+^ retention capacity. Interestingly, Cyclosporine A inhibited Casiopeinas-induced mitochondrial Ca^2+^ release, which suggests the involvement of the mitochondrial permeability transition pore opening. In addition, Casiopeinas reduced the viability of cardiomyocytes and stimulated the activation of caspases 3, 7, and 9, demonstrating a cell death mitochondrial-dependent mechanism. Finally, the early perfusion of Cyclosporine A in isolated hearts decreased Casiopeinas-induced dysfunction with reduction of their toxic effect. Our results suggest that heart cardiotoxicity of Casiopeinas is similar to that of Doxorubicin, involving heart mitochondrial dysfunction, loss of membrane potential, changes in energetic metabolites, and apoptosis triggered by mitochondrial permeability.

## 1. Introduction

The search for cancer treatments has generated novel discoveries of underlying carcinogenesis mechanisms, including discoveries in cardiovascular research, given that many of the targets explored in tumors play critical roles in the heart. The collective efforts of cardiovascular and cancer researchers along with that of clinical researchers are needed to understand how to safely translate such efforts from the laboratory bench to the patients. For instance, anthracyclines such as Doxorubicin (Doxo) can generate heart failure (HF) and left ventricular dysfunction in a dose-dependent manner [1]. Anthracyclines produce cardiac toxicity by increasing myofibrillar disarray and mitochondrial dysfunction [2]. Moreover, Doxo induces reactive oxygen species (ROS) production in the heart via redox cycling of the drug at complex I of the electron transport chain [3]. Therefore, prior work supports the idea that mitochondria are a primary target of both acute and chronic Doxo-induced cardiotoxicity.

Casiopeinas (Cas) are recently developed copper-containing drugs that have shown promising results as chemotherapeutic agents in animal models, as well as in clinical trials [4, 5]. Nevertheless, Cas have shown acute toxicity in a canine model, including cardiac arrhythmias (i.e., bradycardia, heart block, and ventricular arrhythmias) and systolic dysfunction [6]. Cas toxicity has also been related to the inhibition of energy metabolism with changes in glycolytic and oxidative phosphorylation fluxes [7–9]. Experiments in rat hearts have shown that Cas markedly depress contractility and reduce ATP and phosphocreatine (PCr) pools [10]; however, the precise mechanisms behind these responses are still not well understood. Recently, *in vitro* experiments with isolated cardiac mitochondria have shown that Cas increased the oxygen consumption rate at basal respiration and depolarized mitochondrial membrane potential, suggesting that Cas act as mitochondrial uncouplers [8]. In addition, the immunosuppressant cyclosporine A (CsA) inhibited Cas-induced mitochondrial swelling and depolarization, proposing the involvement of the permeability transition pore opening (MPT) [8]. Highlighting its importance, the MPT opening has been associated with matrix swelling and the release of small proapoptotic proteins, such as cytochrome c and oxidative damage of protein or lipids [9, 11]. These changes might be responsible for the inhibitory effect on the electron transport chain in rat heart mitochondria [8] and the Cas-triggered apoptosis observed in neoplastic cells and tumors [12]. In the present study, we aimed to integrate the Cas cardiotoxicity known effects, which include potential impairment of energy metabolism, mitochondrial dysfunction, and MPT involvement, into a whole-heart model, in order to better understand the mechanisms underlying the heart tissue contractile dysfunction and apoptosis. Furthermore, the study explored CsA perfusion as a novel strategy to reduce the toxic effects of Cas in the whole-heart rat model. The proposed mechanisms for Cas were also studied in isolated cardiomyocytes and compared to the effects with Doxo.

## 2. Materials and Methods

### 2.1. Animal Use

All procedures involving animals and their care were performed in accordance with the animal care guidelines of the National Institutes of Health, USA (2011 edition). All procedures were approved by the animal use and care committee of Tecnológico de Monterrey Medical School (protocol number 2011-Re-017).

### 2.2. Ex Vivo Heart Experiments

Male Wistar rats (250–300 g) were injected with heparin (10^3^ U·kg^−1^, i.p.) 20 minutes prior to anesthesia with pentobarbital (100 mg·kg^−1^, i.p.). When bilateral corneal reflex was absent, the heart was excised through an abdominal approach. Then, the ascending aorta was visualized and cut and the heart was placed in a cardioplegic solution. To avoid ischemia, the time between cutting the diaphragm to placing the heart in the solution was less than 60 seconds. The hearts were mounted in accordance with the Langendorff model and perfused at a constant flow (12 ml·min^−1^) with a Krebs-Henseleit buffer as previously described [13]. A latex balloon connected to a pressure transducer filled with saline solution was inserted into the left ventricle after establishing autonomous contraction. The pulmonary artery was cannulated and connected to a closed chamber using a Clark-type oxygen electrode (Yellow Springs Instruments, Yellow Springs, Ohio) to measure myocardial oxygen consumption (MVO_2_) in the coronary effluent. The rate of MVO_2_ was calculated as the difference between the concentration in the K-H buffer before (100%) and after perfusion. Data Trax software (WPI, Sarasota, Florida) was used for continuous recording of the heart rate (HR), left ventricular pressure (LVP), MVO_2,_ and maximum positive and negative derivative of left ventricular pressure (±dP/dt). The baseline was established during 10–15 minutes of K-H perfusion. Each concentration of Cas or Doxo remained in the heart for 30 min. Hearts in the control group were perfused with K-H buffer during the whole experimental time. Rate pressure product (RPP = HR × LVP) and cardiac efficiency (CE = RPP × MVO_2_) were calculated as previously reported [14].

### 2.3. Cell Fractionation and Sample Preparation

To measure the energetic metabolites ATP, phosphocreatine (PCr), and aconitase activity at the end of the perfusion protocols, samples were prepared as follows: the hearts were removed from the perfusion system by cutting the aorta and immediately flash-frozen with liquid N_2_, weighed, and stored at −80°C. Afterward, the hearts were homogenized in ice-cold buffer containing 250 mM sucrose, 10 mM HEPES, and 1 mM EDTA (pH 7.4). For enzymatic determination, aliquots of the homogenate were immediately frozen in liquid nitrogen and stored at −80°C. Quantification of ATP and PCr was carried out in a HPLC system with a dual-pump gradient (Waters Chromatography, Toronto, Canada) as previously described [15].

To measure mitochondrial oxygen consumption, mitochondrial membrane potential, and Ca^2+^ uptake, cytochrome c samples were prepared as follows: after perfusion, heart tissue from the left ventricle was minced and homogenized in cold mitochondrial isolation medium (in mM: 125 KCl, 1 EDTA, and 10 HEPES-HCl; pH 7.3). The mitochondrial fraction was obtained by differential centrifugation using the protease Nagarse, as previously described [16]. Mitochondrial oxygen consumption was measured using a Clark-type oxygen electrode. The experiments were carried out in respiration assay medium containing 125 KCl, 10 HEPES-HCl, and 3 KH_2_PO_4_ (in mM) with pH 7.3. State 4 respiration was measured in the presence of 10 mM glutamate-malate, and state 3 respiration was evaluated after addition of 100 *μ*M ADP. Maximal respiration was determined with 0.08 *μ*M of carbonyl cyanide m-chlorophenyl hydrazine (CCCP) [17]. The mitochondrial membrane potential was measured by fluorometry using 5 *μ*M safranine [13]. Mitochondrial Ca^2+^ uptake was determined with the metalochromic indicator, Arsenazo III, or calcium green 5N (5N-CG) [18, 19], using a medium containing 50 *μ*M Arsenazo or 10 *μ*M 5N-CG, 10 mM succinate plus rotenone (0.1 *μ*g·ml^−1^), 200 *μ*M ADP, and 0.25 *μ*g oligomycin A. Pulses of 10 nmol Ca^+2^ were added every 3 minutes to reach a Ca^+2^ release due to MPT opening. Mitochondrial cytochrome c quantification from Cas-treated hearts was performed using Western blot analysis as described previously [11]. The blots were developed with Luminata crescendo substrate (Merck Millipore, Darmstadt, Germany) and detected with VisionWorks LS-UVP Chimio System (BioSpectrum 415 Imaging System, Cambridge, UK).

### 2.4. Cardiomyocytes Experiments

Ventricular cardiomyocytes were obtained from male Wistar rats (250–300 g) by digestion with collagenase type II as previously described [20]. Cells were washed in Tyrode solution (mM): 130 NaCl, 5.4 KCl, 0.4 NaH_2_PO_4_, 0.5 MgCl_2_, 25 HEPES, and 5 glucose with pH 7.4. After isolation, cells were cultivated at a density of 3.2 × 10^3^ viable cells/well in 96-well plates pretreated with laminin in M-199 medium supplemented in mM with 5 taurine, 5 creatine, 2 L-carnitine, 2.5 sodium pyruvate, and penicillin-streptomycin at 100 U·ml^−1^ and 100 *μ*g·ml^−1^, respectively, at 37°C, with 5% CO_2_ and 95% air. After incubation for three hours, varying concentrations of Cas were tested in triplicate, exposing the myocytes during 24 hours to each treatment. At the end of the incubation period, the cytotoxicity of Cas was measured using the Alamar Blue viability test (Life Technologies, Carlsbad, CA, USA). Release of cytoplasmic lactate dehydrogenase (LDH) was determined using CytoTox-ONE homogeneous membrane integrity kit (Promega Madison, WI, USA). The activity of caspases 3/7 and caspase 9 was measured in cell lysates using caspase-Glo 3/7 and caspase-Glo 9 test luminescent assay (Promega, Madison, WI, USA), respectively. The cardiomyoblast cell line H9c2 (ATCC CRL-1446) was maintained using standard procedures in DMEM supplemented with 10% fetal bovine serum prior to the oxidative stress experiments.

### 2.5. Oxidative Stress Markers

Mitochondrial aconitase activity was measured in isolated heart homogenates by spectrophotometry as previously described [8]. Membrane lipid peroxidation was detected by the TBARS assay using thiobarbituric acid-reactive species as reported by Silva-Platas et al. [8]. Free thiol content was determined by Ellman's reagent, 5,5′-dithiobis(2-nitrobenzoic acid) (DTNB) as previously reported [8]. Thiol groups in the adenine nucleotide translocase were measured by eosin 5-maleimide interaction (EMA) with cysteine residues susceptible to oxidative stress or oxidizing agents [21] and followed by SDS/PAGE electrophoresis. Adenine nucleotide translocase measurement was performed using primary antibody anti-adenine nucleotide translocase (Abcam, MA, USA). The protein bands were detected with secondary HRP conjugated antibody, and the blots were developed as described above. Experiments for anion superoxide production were performed in intact cardiomyoblasts using the fluorescent probe MitoSOX (Molecular Probes) in the flow cytometer. H9c2 cells were loaded with MitoSOX during 15 min at 37°C with a cell density of 100,000 cells/mL (final concentration of MitoSOX 5 *μ*M). Afterwards, the cells were subjected to Cas III-Ea treatment (4 or 20 *μ*M); DOXO (5 *μ*M) and Antimycin A (10 *μ*g/mL) were used as positive controls. Finally, the cells were washed and analyzed on a FACSCanto II (BD Biosciences). FACS data was analyzed using FlowJo version 10.0 (Tree Star).

### 2.6. Chemicals

**Figure 1:**
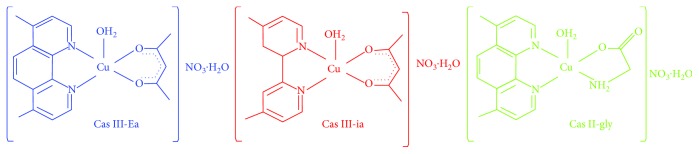
Structures of Cas III-Ea, III-ia, and II-gly.

All chemical reagents were acquired from Sigma-Aldrich (St. Louis, MO, USA), unless otherwise specified. Casiopeina II-gly [Aqua(4,7-dimethyl-1,10-phenanthroline)(glycinate)copper(II)nitrate], Casiopeina III-ia [Aqua(4,4-dimethyl-2,2′bipyridine)(acetylacetonate)copper(II)nitrate], and Casiopeina III-Ea [Aqua(4,7-dimethyl-1,10-phenanthroline)(acetylacetonate)copper(II)nitrate] were synthesized following the synthesis as formerly described [22]. The Cas are referred to as III-Ea, III-ia, and II-gly, respectively. The detailed chemical structure of Cas is shown in Figure 1.

### 2.7. Statistical Analyses of Data

All data were expressed as mean ± SEM. Data were analyzed by ANOVA followed by Dunnett's or Tukey's multiple comparisons test when appropriate using SigmaPlot 10 (Systat Software Inc., Germany) or GraphPad Prism 5 (V.5.01; La Jolla, CA, USA). A *p* value < 0.05 was considered statistically significant.

## 3. Results

### 3.1. Cas Inhibit Cardiac Function in a Time- and Dose-Dependent Manner

**Figure 2:**
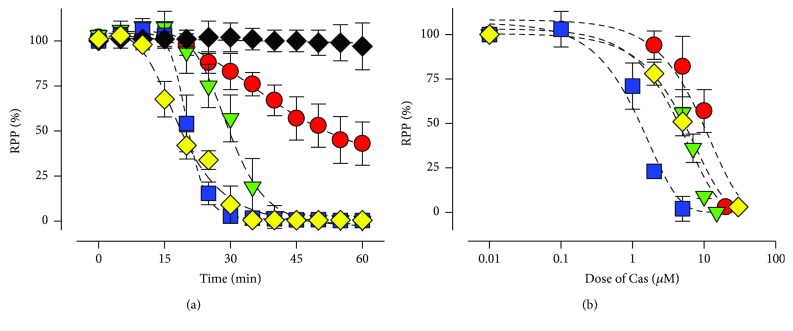
Cas affect contractility on isolated rat hearts. Cas were perfused at 5 *μ*M for 60 minutes (a) or in a dose-dependent manner (b). The blue squares indicate III-Ea, green triangles indicate II-gly, and red circles indicate III-ia. Black rhombuses indicate control and yellow rhombuses indicate Doxo treatment. Values are mean ± SEM. (*n* = 5 experiments at least for each treatment).

To assess the effect of Cas on cardiac function, isolated rat hearts were perfused with equimolar concentrations (5 *μ*M) of III-Ea, III-ia, II-gly, and Doxo. The progress of the RPP on III-Ea perfused hearts was gradually reduced 48% and 97% from the baseline values at 20 and 30 min, respectively (Figure 2(a)). III-Ea and II-gly showed a similar time-dependent inhibitory effect on RPP; however, the inhibitory effect of III-ia was 2.3-fold slower than that of III-Ea. Remarkably, III-Ea exhibited a *t*_0.5_ similar to that of Doxo (Table 1). III-Ea, II-gly, and III-ia induced a dose-dependent progressive and sustained inhibition of RPP, with a half-maximal inhibitory concentration (IC_50_) of 1.3 ± 0.2, 5.5 ± 0.5, and 10 ± 0.7 *μ*M, respectively (Figure 2(b)). II-gly presented a similar IC_50_ to Doxo, and surprisingly, III-Ea showed 3.8-fold more potent effect on RPP than Doxo did (Table 1). As seen in Supplemental Figure 1, the heart rate (HR) was not affected by treatment with III-ia, II-gly, or III-Ea, while Doxo-treatment significantly reduced the HR by 24%. At IC_50_ for RPP, III-ia, III-Ea, and Doxo decreased cardiac efficiency (CE) by 48%, 47%, and 55%, respectively, whereas II-gly lightly impaired CE, causing only a 23% drop (Table 2). In our experimental conditions, all Cas affected the contraction rate (+dP/dt) in a similar manner (≈43%). Furthermore, −dP/dt was inhibited around 70–79%, suggesting a more profound impact on relaxation over contraction.

### 3.2. Cas Impair Energetic Metabolism

**Figure 3:**
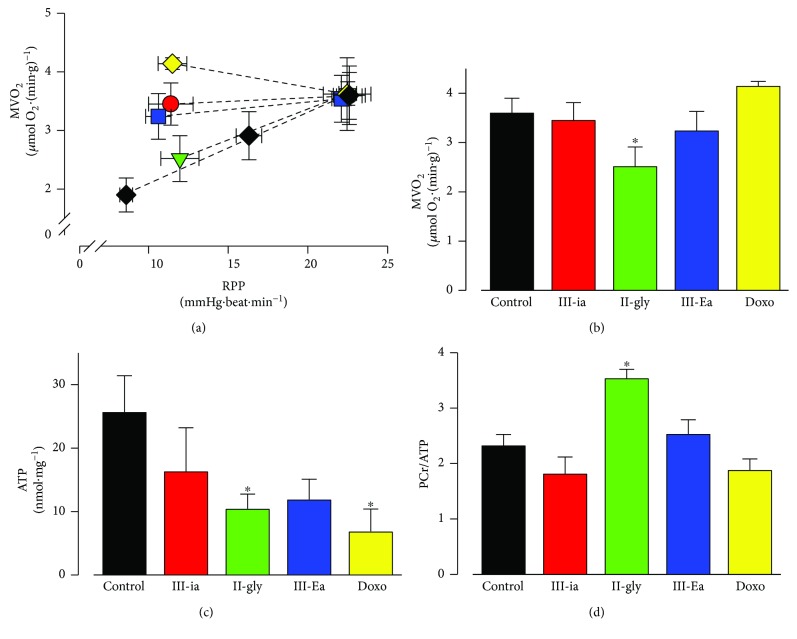
Cas impair oxygen consumption and energetic metabolites. Isolated rat hearts were used to measure the effect of Cas IC_50_ and Doxo (5 *μ*M) on mechanical (RPP) and metabolic coupling (oxygen consumption (MVO_2_) relationship) (a), MVO_2_ (b), ATP content (c), and myocardial PCr/ATP ratio (d). Values are mean ± SEM. ^∗^ *p* < 0.05 versus control (*n* = 5 experiments at least for each treatment).

It is well known that under normal conditions, the relationship between RPP and MVO_2_ should be linear, given that any increase in cardiac contractility should be accompanied by a proportional increase in MVO_2_ (ATP production in control conditions).  Figure 3(a) displays this linear relationship between RPP and MVO_2_. Interestingly, Cas-treated hearts showed a reduced slope, thus evidencing an apparent inefficient coupling between contraction and O_2_ consumption. III-ia, III-Ea, and Doxo required, respectively, a 1.45-, 1.47-, and 1.8-fold additional MVO_2_ to generate the same contractile force compared to the control hearts. Nevertheless, II-gly had a RPP/MVO_2_ ratio similar to the control. There were no changes in MVO_2_ with III-ia, III-Ea, or Doxo treatments; however, a 37%, 56%, and 74% decrease, respectively, in ATP content was observed (Figures 3(b) and 3(c)). Thus, III-ia, III-Ea, and Doxo act similarly to CCCP (a mitochondrial uncoupler), which stimulates MVO_2_ by uncoupling ATP synthesis from the mitochondrial electron transport [23]. III-ia and Doxo also decreased PCr levels. Furthermore, the PCr/ATP ratio was augmented only in II-gly-treated hearts (Figure 3(d)), showing a PCr accumulation, which might point to inability of the myofibrillar creatine kinase system to rephosphorylate ADP.

### 3.3. Cas Uncouple Mitochondrial Respiratory Chain and Induce Permeability Transition Pore

**Figure 4:**
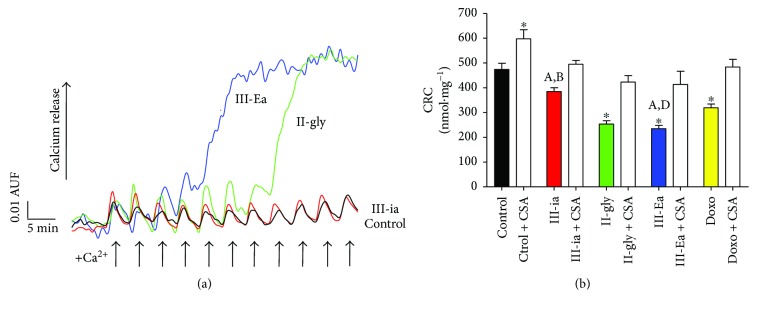
Cas treatments induce MPT opening. (a) Representative recording of Ca^2+^ retention capacity (CRC) experiment with isolated mitochondria from IC_50_ Cas-treated and Doxo (5 *μ*M) hearts and (b) semiquantitative analysis of mitochondrial CRC in the presence of CsA (0.5 *μ*M). Arrows indicate 10 *μ*M pulses of Ca^2+^. Values are mean ± SEM. ^∗^ *p* < 0.05 versus control; *p* < 0.05 versus (A) Doxo, (B) II-gly, and (D) III-ia (*n* = 5 animals for each treatment, exception Doxo groups (*n* = 3)).

To explore the effect of Cas on mitochondrial function, the respiratory activities of mitochondria isolated from Cas-treated hearts were measured. NADH-linked respiratory rate was determined using malate/glutamate as a substrate (Table 3). Mitochondria from II-gly- and III-ia-treated hearts exhibited a 77% and 78% decrease in the state 3 respiration rates, respectively. Consistently, an 82% and 87% reduction in maximal respiration was revealed, respectively, suggesting a potent inhibition of the respiratory chain. On the other hand, a moderate inhibitory effect on maximal mitochondrial respiration and an increased state 4 respiration rate was shown in mitochondria from III-Ea-treated hearts compared to III-ia- and II-gly-treated ones. However, the respiratory control ratio was depressed in mitochondria from hearts treated with III-Ea, indicating an uncoupling effect. In addition, to determine whether a Cas-induced MPT opening was evident, mitochondrial Ca^2+^ retention capacity experiments were performed. As shown in Figures 4(a) and 4(b), the mitochondrial Ca^2+^ retention capacity of III-Ea-treated hearts decreased by 50.5%, while that of II-gly decreased by 46.5% compared with the control. A protective effect of CsA (*in vitro* addition) treatment was also evident, as shown in Figure 4(b). CsA significantly protected Cas and Doxo effects. Mitochondria from III-Ea-treated hearts in the presence of CsA showed a 1.8-fold delay in the opening of MPT. In mitochondria treated by III-ia, II-gly, and Doxo, CsA demonstrated a protective effect of 28%, 60%, and 51%, respectively.

### 3.4. Effect of Cas on Mitochondrial ROS and TBARS Production

**Figure 5:**
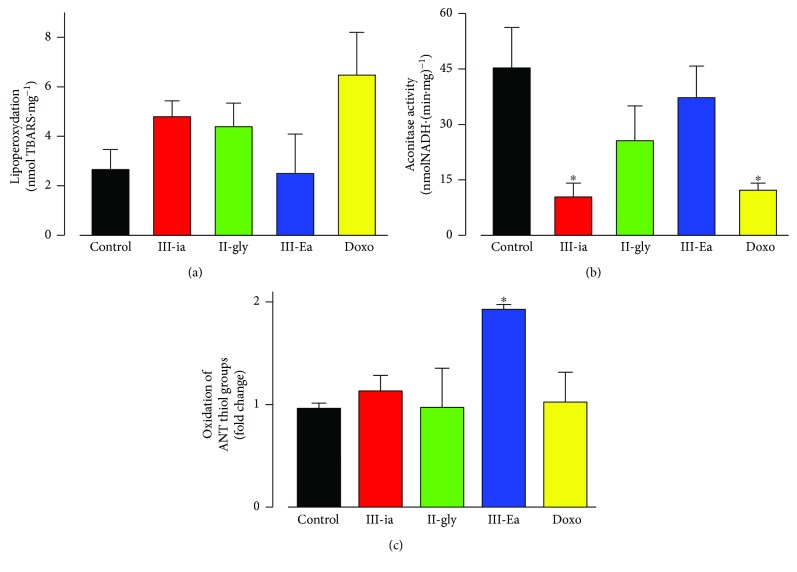
Effect of Cas on isolated heart on TBARS content (a), aconitase activity (b), and ANT/thiols groups (c) on mitochondria from IC_50_ Cas-treated and Doxo (5 *μ*M) hearts. Values are mean ± SEM. ^∗^ *p* < 0.05 versus control (*n* = 5 animals for each treatment, except for Doxo group (*n* = 3)).

Mitochondrial activity, aconitase activity, and TBARS content were measured in isolated hearts to determine the role of oxidative damage of Cas in triggering mitochondrial dysfunction. In the ex vivo heart, only III-ia decreased aconitase activity, an effect that was also observed in the Doxo-treated hearts (Figure 5(b)). Thiol groups and specific thiol groups in adenine nucleotide translocase from mitochondria isolated from Cas-treated hearts were measured. As shown in Figure 5(a), there was no increase in lipid peroxidation, nor in thiol oxidation (data not shown) with the Cas treatments (Figure 5(a)) when compared with the control. In addition, mitochondrial anion superoxide production was measured using the MitoSOX probe. Histograms of flow cytometry analysis (Supplemental Figure 2A) showed a 9-fold increase in mean intensity in cells treated acutely with Antimycin A (a well-known mitochondrial inhibitor and inducer of anion superoxide production) and a 4-fold increase with Doxo. However, there was no change in the anion superoxide production in intact cells with III-Ea treatment at 4 or 20 *μ*M, suggesting that mitochondrial ROS production is not significant in III-Ea cell injury (Supplemental Figure 2B). On the other hand, there was a twofold increase in the oxidation state of adenine nucleotide translocase thiol groups with the III-Ea treatments (Figure 5(c)), an effect previously reported in isolated mitochondria [8]. It has been shown that o-phenanthroline, in addition to Cu^+2^, interacts directly with adenine nucleotide translocase inducing a cytosolic-conformational state, which inhibits ADP/ATP translocation and rises MPT opening [15]. In III-Ea, Cu^+2^ is coordinated with 4,7-dimethyl-1,10-phenanthroline. It thus seems that the interaction with adenine nucleotide translocase, which increased the thiol group oxidation and the MPT opening, does not require ROS production and oxidative damage. In summary, the observed effects of Cas in the isolated heart model were not strong enough to induce significant phospholipid or protein damage.

### 3.5. Cas Trigger Mitochondrial Apoptosis

**Figure 6:**
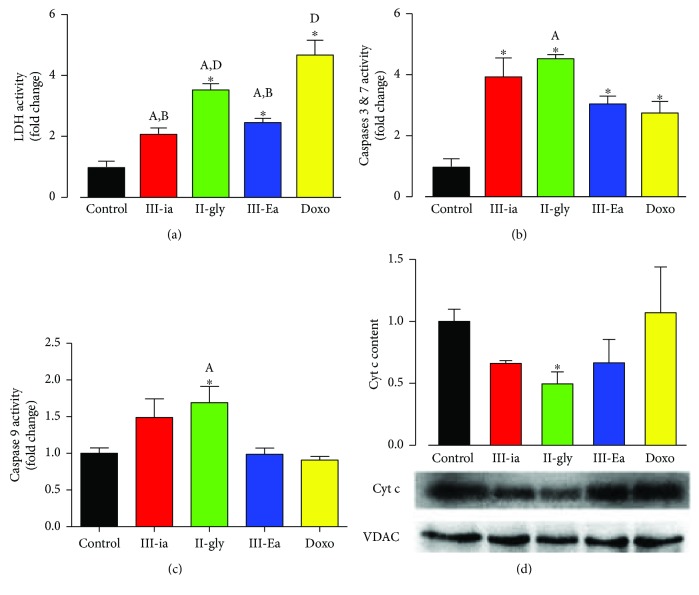
Cas trigger mitochondrial cell death. Panels (a–c) shows LDH, caspase 3/7 and caspase 9 activities on isolated cardiomyocytes treated with Cas at its IC_50_ (in *μ*M: III-ia (7), II-gly (2), III-Ea (2), and Doxo (10)). Panel (d) shows cytochrome c content by Western blot analysis in heart mitochondria after Cas-perfusion for 30 minutes in the ex vivo hearts at its IC_50_ (in *μ*M: III-ia (10), II-gly (5.5), III-Ea (1.3), and Doxo (5)). Values are mean ± SEM. ^∗^ *p* < 0.05 versus control; *p* < 0.05 versus (A) Doxo, (B) II-gly, and (D) III-ia (*n* = 5 experiments for each treatment, except for panel (d) (3 animals for group)).

To assess the effect of Cas on cardiomyocyte viability, we cultured adult rat ventricular myocytes with different concentrations of Cas (0–1000 *μ*M) and examined cell death after a 24 h treatment using the Alamar blue assay. As shown in Supplemental Figure 3, III-Ea, II-gly, and III-ia induced significant cytotoxic effects in a dose-dependent manner, with a LD_50_ of 2 ± 0.4, 2 ± 0.5, and 7 ± 1.7 *μ*M, respectively, indicating a 3-fold higher toxicity of III-Ea and II-gly compared to III-ia. These results are consistent with previous quantitative structure-activity relationship (QSAR) studies [4]. Likewise, IC_50_ from III-Ea was 5-fold higher than that of Doxo (10 ± 0.5 *μ*M). At its IC_50_, Cas induced diverse cytotoxic effects and cell death mechanisms, evidenced by different release levels of LDH, a marker of necrosis (Figure 6(a)). Doxo and II-gly produced a 4.7- and 3.5-fold increase in LDH release, respectively, compared to the control. On the other hand, activity of caspases 3 and 7 increased ~4-fold for III-ia and II-gly treatments, compared to Doxo or the control. Interestingly, the significant increases in caspase 3 and 7 activities observed for III-ia and II-gly treatments correlated directly with reduction of cytochrome c from the mitochondria, suggesting the activation of a mitochondria-dependent apoptosis pathway (*p* < 0.5) (Figure 6(d)). However, for caspase 9, only II-gly caused a 1.7-fold significant (*p* < 0.05) increase in activity (Figure 6(c)).

### 3.6. Early Perfusion of CsA in Isolated Hearts Ameliorates the Cas Effect due to MPT Opening

**Figure 7:**
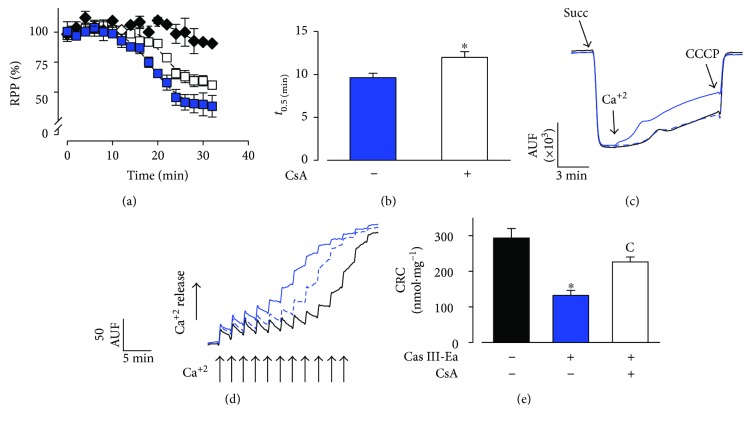
Early perfusion of CsA in isolated hearts ameliorates the Cas effect due to MPT opening. RPP is shown in (a). The decline in contractility due to the perfusion (20 min) of 10 *μ*M III-Ea alone is presented as the blue trace. The early perfusion (10 min) of 1 *μ*M CsA (white squares) delays the decline in contractility due to the subsequent perfusion with III-Ea. The control trace is presented as the black trace. Analysis of the traces is presented as the time for half inhibition (*t*_0.5_) (b). Experiments in isolated mitochondria prepared from these hearts at the end of perfusion. Representative recording of membrane potential (c) and Ca^2+^ retention capacity (d). Semiquantitative analysis of mitochondrial CRC (e). Mitochondria from III-Ea-treated hearts are represented as a blue solid line, mitochondria from CsA-Cas III-Ea hearts as a blue dot line, and untreated hearts as a black solid line. Arrows indicates succinate (10 mM), CCCP (0.08 *μ*M), or 10 *μ*M pulses of Ca^2+^ addition. Values are mean ± SEM. ^∗^ *p* < 0.05 versus control; *p* < 0.05 versus (C) III-Ea (*n* = 4 animals for each treatment).

To prove the role of MPT opening on the Cas-mediated cardiotoxicity, experiments were performed in the whole-heart model treated with CsA. As previously described, 1 *μ*M CsA was perfused during 10 minutes, then 10 *μ*M III-Ea was perfused while recording the left ventricular pressure to calculate the RPP and evaluate mechanical performance. As expected, the perfusion of III-Ea resulted in a decline of contractility (*t*_0.5_ of 9.65 min). However, treatments with the perfusion of CsA prior to III-Ea perfusion resulted in a significant reduction in contractile dysfunction (Figure 7(a)). RPP analyses showed a significantly protective effect (*t*_0.5_ of 11.98 min, *p* = 0.035, *n* = 4) exerted by CsA with respect to the cardiotoxicity observed for III-Ea alone (Figure 7(b)). Results from functionality of heart-isolated mitochondria at the end of perfusion treatments are shown in Figures 7(c), 7(d), and 7(e). A representative recording of membrane potential (Figure 7(c)) showed the depolarization of mitochondria after the addition of 10 *μ*M Ca^+2^, from III-Ea-treated hearts; however, mitochondria from CsA-III-Ea-treated hearts remained polarized similar to mitochondria from untreated hearts. Calcium retention capacity analyses indicated that mitochondria from CsA-III-Ea-treated hearts tolerated 69% more calcium than mitochondria from III-Ea hearts (226 ± 13 versus 133 ± 14 nmol·mg^−1^, *p* = 0.03, *n* = 4). Hence, the uncoupling of contractility and energetics in the hearts treated with III-Ea was observed to be partly dependent on MPT opening.

## 4. Discussion

In recent years, more powerful and specific drugs have been developed to treat cancer. Unfortunately, several highly effective antineoplastic drugs have reported cardiotoxicity as a side effect, including Doxo and trastuzumab, a recombinant humanized antibody [2, 24, 25]. The use of Doxo as an antineoplastic drug has been mostly hampered by some adverse cardiovascular events such as hypertension, ventricular dysfunction, and HF [1, 26]. The onset of these adverse cardiovascular events might take place early or be delayed up to two decades after the conclusion of cancer treatment [1, 27]. Noteworthy, novel chemotherapeutic agents such as Cas have shown more potent antitumoral activity than Doxo [28]. For instance, the reduction of the volume of subcutaneous tumors in nude mice achieved by III-ia was 81% [29] while that of Doxo was 20% [30]. However, in cellular and isolated mitochondrial models, Cas have shown a remarkable cytotoxic effect [8]. In this regard, as an attempt to determine the potential cardiotoxicity of Cas (ex vivo) and its underlying mechanisms, this work examined the acute cardiotoxicity of Cas treatments in ex vivo heart and isolated cardiomyocytes. Our experimental results have shown that 1-10 *μ*M of Cas decline cardiac metabolism and contractility. These events are dose- and time-dependent at similar or lower concentrations than Doxo [1]. Similarly, as previously observed in mice and *in vitro* experiments, III-Ea and II-gly (with phenanthroline substituents) were 7- and 2-fold more potent inhibitors of cardiac contractility, respectively, compared to III-ia (with bipyridine as a ligand) [4]. These results suggest that a decrease in cardiotoxicity and a slower effect on RPP could be attributable to the absence of the third benzene ring in bipyridines. Both ligands in III-Ea, phenanthroline and acetylacetonate, render the compound more permeable than II-gly, whose second ligand is glycinate instead of acetylacetonate, and more permeable than III-ia, whose imine ligand is dipyridine. In both, the whole-heart model and in adult rat cardiomyocytes, III-Ea and II-gly exhibited a lower IC_50_ for RPP and cell viability compared to III-ia. Yet, III-Ea showed a lower IC_50_ than II-gly in the whole-heart model. This might be resultant to the length of the second carbon ligand (acetylacetonate) that also facilitates the early decline of contractility by interrupting the ATP supply. Previous experiments in cardiac mitoplasts compared to intact mitochondria indicated that phenanthroline ligands might accelerate copper transport and cause intracellular and mitochondrial damage [8]. This effect might be accredited to the hydrophobicity of the substituents, which rises passive uptake of copper [22]. Thus, the phenanthroline ligands of III-Ea and II-gly might act as carriers in the cellular membrane transport of copper compounds. On the other hand, the uncoupling effect on mitochondria might depend on the acetylacetonate or glycinate ligand. These substituents in metal coordination compounds have shown an acidic-dissociable acetyl group with a pKa ≈ 6, which corresponds to an electron-withdrawing moiety. This dissociation could stabilize the anionic species through delocalization of the charge over its structure [31]. Subsequently, protonated III-Ea and II-gly could move from the intermembrane space into the mitochondrial matrix, dissociate, and then diffuse back in their ionized form to the intermembrane space, where they might be protonated again, repeating the cycle. However, an acidic-dissociable acetyl group is not enough to account for a mitochondrial uncoupling effect. In addition to the Cas geometric arrangement, the hydrophobicity of phenanthroline is required to transport protons across the inner membrane, possibly through adenine nucleotide translocase, dissipating the membrane potential required for the ATP synthesis (Table 3). Therefore, Cas III-ia, without a phenanthroline substituent, is not prone to produce mitochondrial membrane depolarization and MPT opening (Figure 4). On the other hand, a reactive acetylacetonate ligand, as observed in III-ia and III-Ea, can potentially contribute to the reduction potential of the copper center as a decisive factor in ROS-production. From an experimental point of view, a previous report from our group observed that III-ia induced a twofold decrease in *α*-ketoglutarate dehydrogenase activity compared with III-Ea [8]. Besides, there was no mitochondrial ROS production by III-Ea. These conflicting results could indicate that III-Ea remains preferentially at the mitochondrial inner membrane (interacting with MPT components, such as the adenine nucleotide translocase) instead of the mitochondrial matrix interacting with soluble enzymes such as *α*-ketoglutarate dehydrogenase. However, the molecular mechanism by which the glycinate ligand in II-gly contributes to mitochondrial dysfunction is unclear. II-gly shows a lower depolarizing effect than III-Ea but a similar effect on mitochondrial inhibition as that of III-ia. A II-gly-reduced effect on mitochondrial inhibition might be synergized with the uncoupling effect of the phenanthroline ligand, producing a toxic Cas. In this regard, production of ROS by a Fenton-like reaction consuming reducing metabolites [32] or by generation of stable copper-GSH (reduced glutathione) compounds [12, 32] are the previously described mechanisms of II-gly- and III-ia-induced injury. The reactions in both mechanisms lead to membrane damage and lipid peroxidation [32]. Nevertheless, our experiments in cardiac tissue and cardiomyoblasts indicate that oxidative stress is not enough to completely explain Cas cardiotoxicity. In fact, there is strong evidence that Cas cardiotoxicity arises from ROS-independent mechanisms, such as impairment of cellular energetics, which compromises the cardiomyocyte ability to generate adequate contraction-relaxation cycles. Perhaps the high activity of antioxidant enzymes and the levels of GSH within the heart provide a strong antioxidant defense system against the consequent oxidative stress injury [33]. In brief, our results point to the mitochondria as the main target for Cas cardiotoxicity, impairing oxidative phosphorylation and inducing energetic failure in the whole heart. These findings correlate with the mitochondrial dysfunction observed in mitochondria isolated from Cas-treated hearts. An acute uncoupling effect, collapsing the mitochondrial membrane potential, was the most consistent effect of Cas at both, the whole-heart and the mitochondrial levels. Mitochondria isolated from Cas-treated hearts presented limited ability to conduct ADP-coupled oxidative phosphorylation, given the lower respiratory control (Table 3 and Figure 7(c)). Whole hearts showed induction of the MPT opening, which generated a disruption of the membrane potential and an uncoupling of the respiratory chain, resulting in an increased MVO_2_. In this context, it has been demonstrated that mitochondrial uncouplers induce apoptosis in several cell types [34], supporting the idea that Cas-induced mitochondrial uncoupling causes membrane permeabilization. Increased permeability to protons of the mitochondrial membrane could occur in circumstances of extensive and nonspecific membrane damage, such as those implicated in protein oxidation due to excessive ROS production [35]. Our results did elicit a significant prooxidant action on adenine nucleotide translocase thiol groups in the hearts during III-Ea treatment, although not entirely due to mitochondrial ROS production. Hence, Cas might play a direct role in mitochondrial permeability and mitochondrial-triggered apoptosis. Accordingly, Silva-Platas et al. [8] have observed III-Ea-induced MPT opening and extensive mitochondrial membrane depolarization and swelling [9]. Mitochondrial swelling has been associated with the release of cytochrome c and cell death, particularly in cardiomyocytes [11, 18, 36]. It has been shown that apoptosis induction by Cas acting as uncouplers or MPT openers might be achieved via cytochrome c release followed by the activation of caspases [37, 38]. In accordance, we found that cytochrome c was decreased in mitochondria isolated from Cas-treated hearts (Figure 6(d)), indicating its release out of the mitochondria. On this point, Nakagawa et al. observed that cytochrome c can be released out of the mitochondria via the MPT, which in turn is induced by Ca^2+^, without the involvement of proapoptotic proteins like Bax [39]. We found no significant changes in Bax (data not shown), probably due to the early stages of apoptosis in the Cas-treated hearts. It is also noteworthy that our experiments did not include *in vivo* models, which can alter or change the way Cas exert their effects.

As formerly reported, Cas binding to plasma proteins prompts their different availability between ex vivo and *in vitro* conditions [40], just as has been observed with Doxo [41]. On the other hand, while pharmacokinetics, elimination time, and distribution of Cas are only partially understood [6, 40], the defined therapeutic dose is still a source of debate. However, in the current ex vivo study, there were no plasma proteins such as albumin, which has been found to bind near 80% of the Cas dose [42]. Therefore, a 5-fold higher *in vivo* dose would be needed to achieve current IC_50_ cardiotoxic levels using an acute dose, while it is completely unclear during chronic exposure.

Currently, monitoring and reducing cardiotoxicity of cancer drugs is of the utmost priority. With respect to Cas, the mechanism underlying their cardiac side effects appears to involve the MPT opening, which produces an energetic debacle. Our results demonstrated that the early perfusion of CsA, prior to the exposure to Cas, ameliorated the decline in contractility in isolated rat hearts. MPT opening was the protection mechanism involved, which was also evidenced by the functionality of mitochondria isolated from the perfused hearts. Features of MPT opening and mitochondrial-triggered apoptosis are consistent with previous reports of Doxo-induced cardiomyocyte toxicity [26, 43], in which the CsA effect was also reported to be cardioprotective in the rat heart in a dose-dependent form [37]. In this context, pharmacological inhibition of the MPT opening improved cardiac function by reducing heart injury in animal models and in patients [16, 44]. Also, pretreatment with carvedilol delayed death in Cas-treated dogs [6] acting similarly as CsA, by reducing the MPT opening and the release of cytochrome c [45]. Moreover, the use of CsA has been found to increase the chemosensitivity in non-small lung cancer cells resistant to epidermal growth factor receptor tyrosine kinase inhibitors [46] and cisplatin-resistant ovarian cancer [47]. These effects occur mainly by augmenting STAT3 inhibition in tumor cells. In conclusion, we determined Cas-mediated cardiotoxicity in an acute setting and demonstrated that III-Ea is the most cardiotoxic Cas, since it compromised adenine nucleotide translocase thiol groups, increased the MPT opening, and uncoupled the mitochondrial energetic function of cardiac mechanical performance. The early perfusion of CsA ameliorated the decline in contractility, demonstrating the involvement of MPT opening in III-Ea cardiotoxicity. Further work implicates designing subacute or longer-term studies to fully understand the magnitude and complexity of Cas cardiotoxicity and more thoroughly to elucidate the molecular pathophysiology during a chronic exposure. Our results contribute with new scientific knowledge by identifying a possible mechanism involved in Cas-induced cardiac side effects, which is crucial to the further improvement of more potent and efficacious cancer therapies with less cardiotoxicity.

## Figures and Tables

**Table 1:** Dose- and time-dependent parameters of hearts treated with Cas.

	Cas III-ia (*n* = 6)	Cas II-gly (*n* = 7)	Cas III-Ea (*n* = 8)	Doxo (*n* = 5)
IC_50_ (*μ*M)	10 ± 0.7^a,b,c^	5.5 ± 0.5^b,d^	1.3 ± 0.2^a,b,d^	5 ± 0.3^c,d^
*t*_0.5_ (min)	49 ± 0.8^a,b,c^	29 ± 1.0^d^	21 ± 5.0^d^	18.6 ± 4.0^d^

The time for half-inhibition (*t*_0.5_) at 5 *μ*M and the half-maximal inhibitory concentration (IC_50_) at 30 min of Cas-treated hearts. Values are mean ± SEM. *p* < 0.05 versus ^a^Doxo, ^b^II-gly, ^c^III-Ea, and ^d^III-ia (*n* = 5 experiments at least for each treatment).

**Table 2:** Effect of Cas on cardiac efficiency, contraction, and relaxation rates.

	Control (*n* = 9)	Cas III-ia (*n* = 6)	Cas II-gly (*n* = 7)	Cas III-Ea (*n* = 8)	Doxo (*n* = 5)
Cardiac efficiency (RPP · MVO_2_^−1^)	6.2 ± 0.25	3.2 ± 0.4^∗^	4.8 ± 0.81	3.3 ± 0.2^∗^	2.8 ± 0.1^∗^
+dP/dt × 1000 (mmHg·s^−1^)	4.2 ± 0.41	2.4 ± 0.20^∗^	2.0 ± 0.13^∗^	2.4 ± 0.23^∗^	2.1 ± 0.23^∗^
−dP/dt × 1000 (mmHg·s^−1^)	−3.3 ± 0.22	−1.0 ± 0.16^∗^	−0.7 ± 0.09^∗^	−0.8 ± 0.13^∗^	−0.8 ± 0.18^∗^

Rat hearts were treated with Cas or Doxo at the IC_50_. CE: cardiac efficiency; RPP: rate-pressure product; MVO_2_: myocardial oxygen consumption; +dP/dt: contraction rate; −dP/dt: relaxation rate. Values are mean ± SEM. ^∗^ *p* < 0.05 versus control (*n* = 5 experiments at least for each treatment).

**Table 3:** Mitochondrial respiratory activity from Cas-treated hearts.

	Control (*n* = 5)	Cas III-ia (*n* = 3)	Cas II-gly (*n* = 3)	Cas III-Ea (*n* = 3)	Doxo (*n* = 4)
State 3 respiration (nmol O min^−1^·mg^−1^)	86.17 ± 7.5	18.5 ± 0.6^∗^	20.2 ± 7.3^∗^	37.3 ± 2.7^∗^	25 ± 8.8^∗^
State 4 respiration (nmol O min^−1^·mg^−1^)	25.5 ± 4.4	15.6 ± 2.6	13.0 ± 5.0	24.5 ± 5.4	18 ± 2.0
Max. respiration (nmol O min^−1^·mg^−1^)	90.1 ± 9.0	12.3 ± 2.6^∗^	16.5 + 3.0^∗^	48.3 ± 5.7^∗^	25 ± 15.1^∗^
Respiratory control	4.1 ± 0.7	1.3 ± 0.1^∗^	1.6 ± 0.3^∗^	1.5 ± 0.2^∗^	1 ± 0.3^∗^

Values are mean ± SEM. ^∗^ *p* < 0.05 versus control.

## Supplementary Materials

Supplementary 1 Figure 1: heart rate (HR) was not affected by the Cas treatment. HR was measured in rat heart perfused with Cas at 5 *μ*M. Values are mean ± SEM. ^∗^ *p* < 0.05 versus control (*n* = 5 experiments at least for each treatment).

Supplementary 2 Figure 2: anion superoxide production in the myoblast cell line H9c2 exposed to (*μ*M) Doxorubicin (5), III-Ea (4 and 20), and Antimycin A (10 *μ*g/mL) using the fluorescent probe MitoSOX and flow cytometry. The representative histogram of the MitoSOX signal is presented in A, and the mean fluorescence intensity is calculated and compared in B. ^∗^ *p* < 0.05, *n* = 3.

Supplementary 3 Figure 3: dose-dependent effect of Cas (0–1000 *μ*M) on cardiomyocyte viability. Values are mean ± SEM (*n* = 5 experiments at least for each treatment).
