# Supplementary figures and images for: Ex Vivo Cardiotoxicity of Antineoplastic Casiopeinas Is Mediated through Energetic Dysfunction and Triggered Mitochondrial-Dependent Apoptosis
Source: Oxid Med Cell Longev. 2018 Mar 25;2018:8949450. doi: 10.1155/2018/8949450 (PMC5889877; doi:10.1155/2018/8949450)

Supplemental figure 1

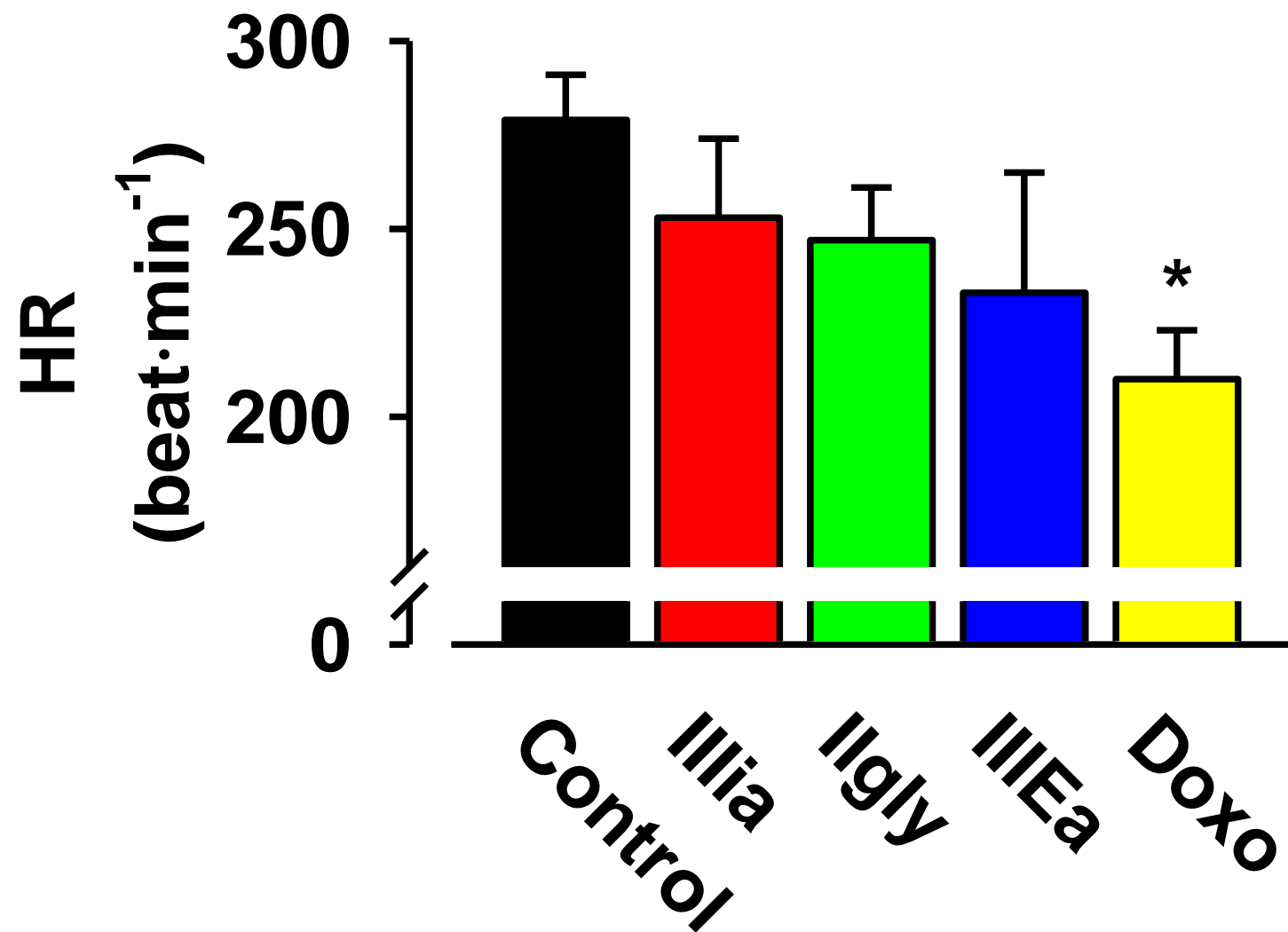

Supplement: Supplementary 1 — Figure 1: heart rate (HR) was not affected by the Cas treatment. HR was measured in rat heart perfused with Cas at 5 μM. Values are mean ± SEM. ∗ p < 0.05 versus control (n = 5 experiments at least for each treatment). [file 8949450.f1.pdf]

Supplemental figure 2

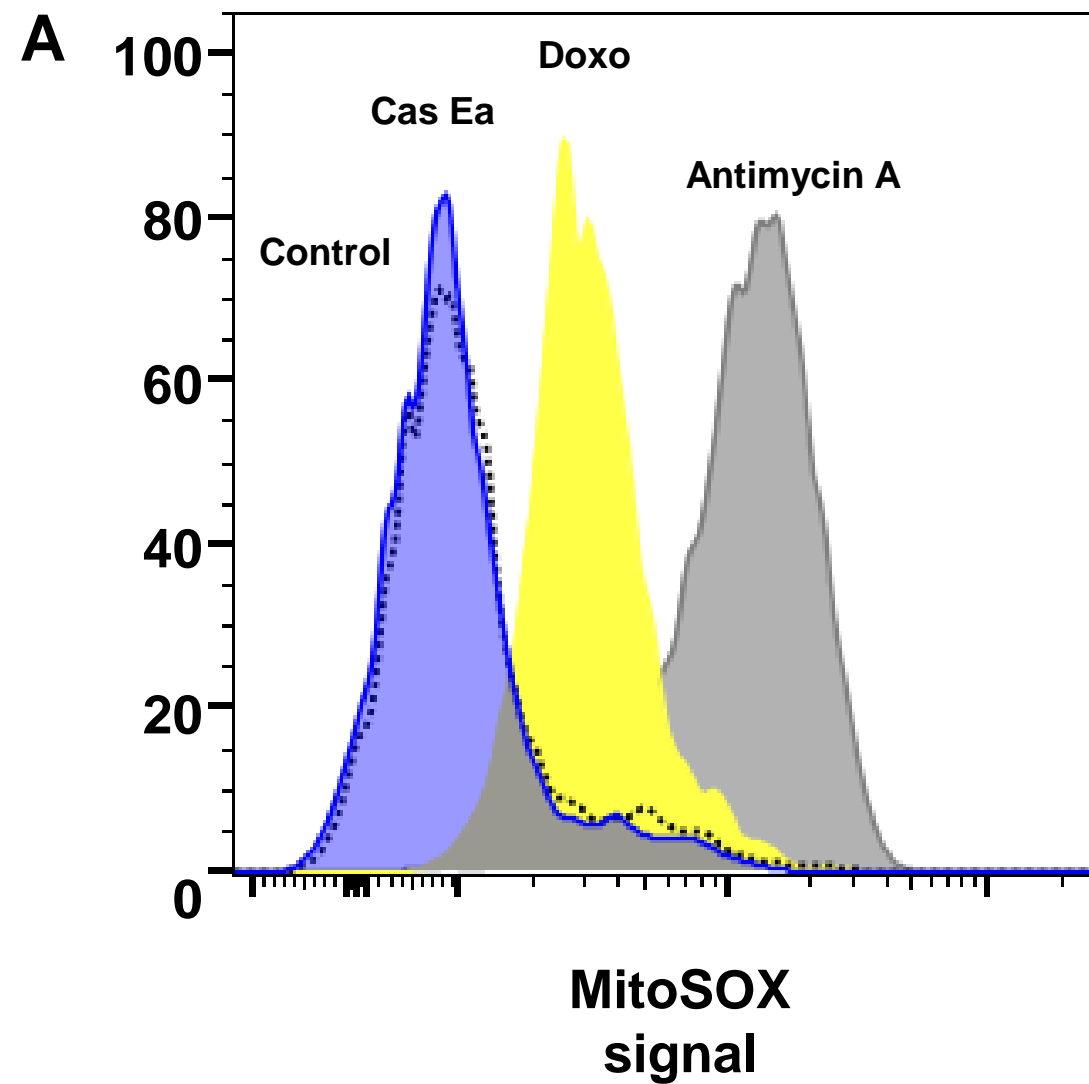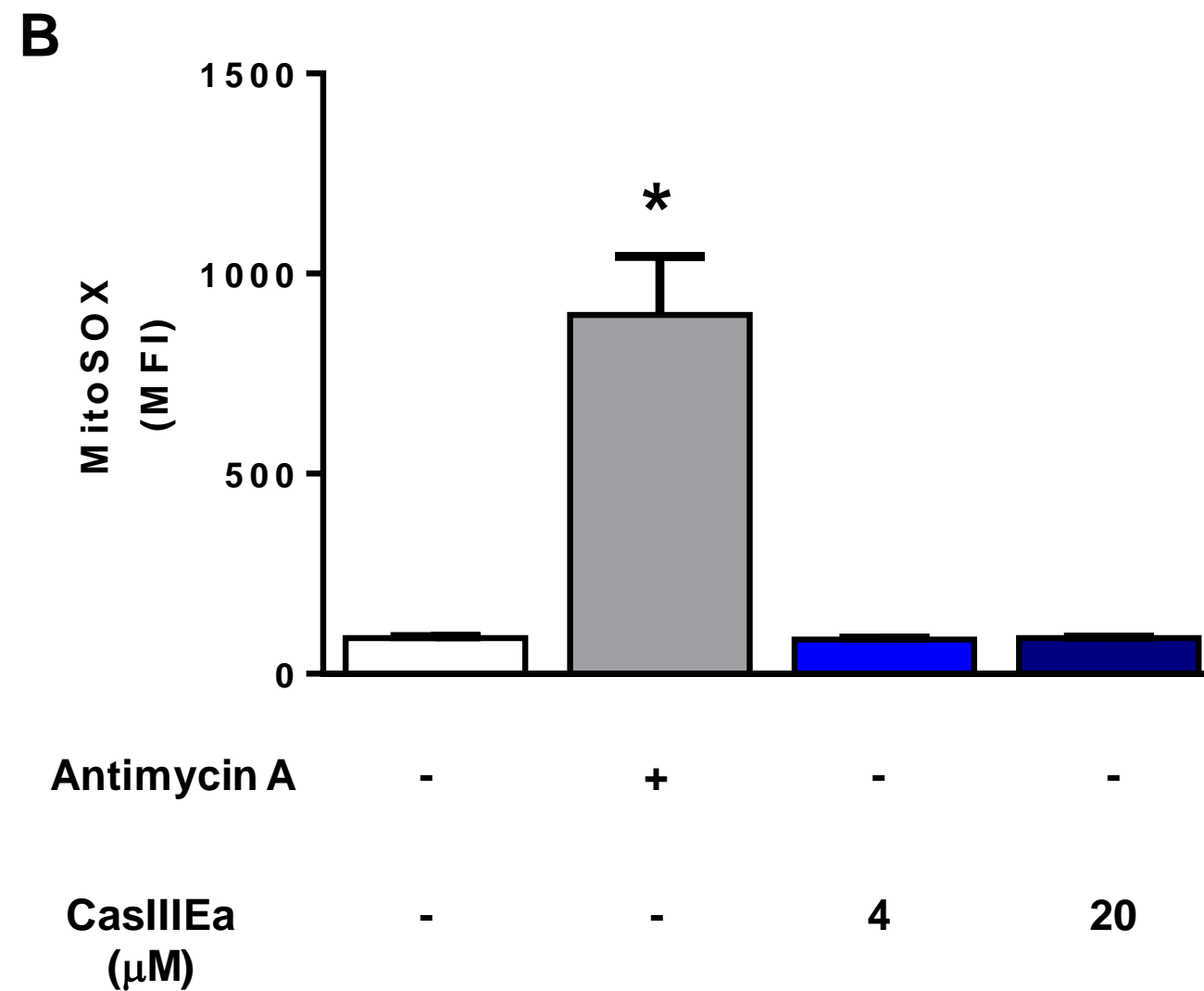

Supplement: Supplementary 2 — Figure 2: anion superoxide production in the myoblast cell line H9c2 exposed to (μM) Doxorubicin (5), III-Ea (4 and 20), and Antimycin A (10 μg/mL) using the fluorescent probe MitoSOX and flow cytometry. The representative histogram of the MitoSOX signal is presented in A, and the mean fluorescence intensity is calculated and compared in B. ∗ p < 0.05, n = 3. [file 8949450.f2.pdf]

Supplemental figure 3

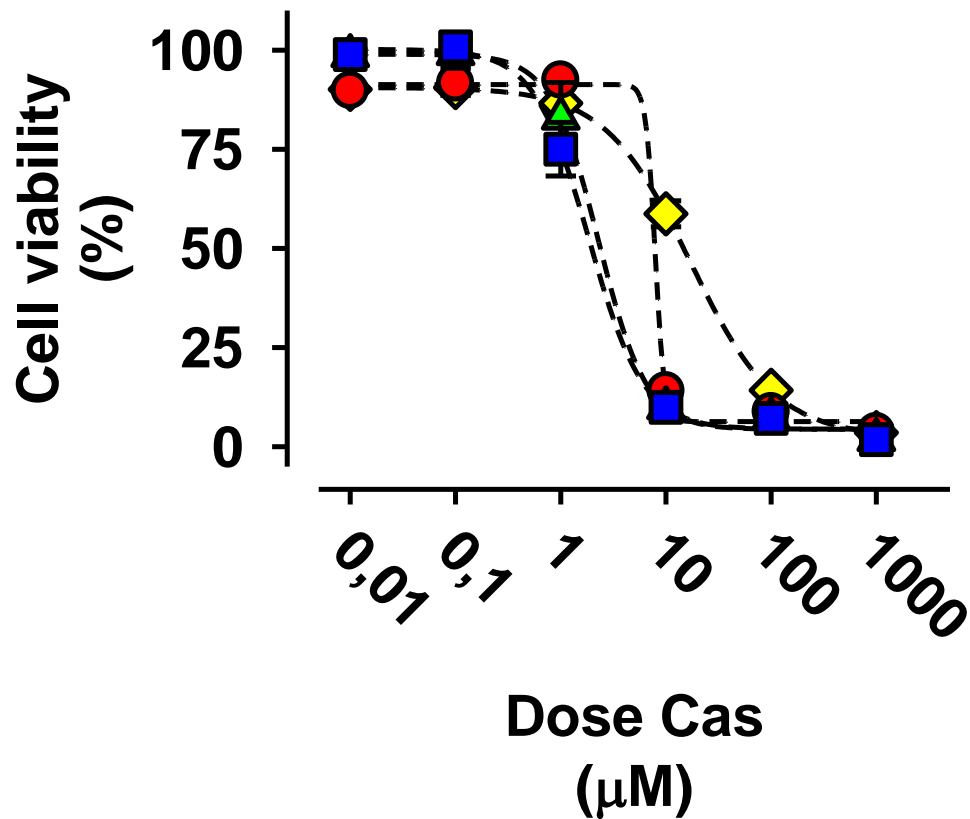

Supplement: Supplementary 3 — Figure 3: dose-dependent effect of Cas (0–1000 μM) on cardiomyocyte viability. Values are mean ± SEM (n = 5 experiments at least for each treatment). [file 8949450.f3.pdf]
